# The mechanism of metal exchange in non-metallic nanoclusters†

**DOI:** 10.1039/c9na00746f

**Published:** 2020-01-14

**Authors:** Shuxin Wang, Lin Xiong, Guodong Sun, Li Tang, Jun Zhang, Yong Pei, Manzhou Zhu

**Affiliations:** Department of Chemistry, Center for Atomic Engineering of Advanced Materials, Anhui University Hefei Anhui 230601 PR China ixing@ahu.edu.cn zmz@ahu.edu.cn; Department of Chemistry, Key Laboratory of Environmentally Friendly Chemistry and Applications of Ministry of Education, Xiangtan University Xiangtan Hunan 411105 PR China ypnku78@gmail.com; School of Materials and Chemical Engineering, Anhui Jianzhu University Hefei Anhui 230601 PR China

## Abstract

We substituted gold atoms in fcc structured Au_28_ and Au_36_ nanoclusters with a Ag(i)SR complex and obtained Ag_*x*_Au_28−*x*_ and Ag_*x*_Au_36−*x*_ nanoclusters, respectively. The positive electrostatic potential (ESP) and dual descriptor (Δ*f*) values were calculated for the metal cores of both nanoclusters, which indicated that the metal exchange is an electrophilic reaction.

## Abstract 2

We substituted gold atoms in fcc structured Au_28_ and Au_36_ nanoclusters with a Ag(i)SR complex and obtained Ag_*x*_Au_28−*x*_ and Ag_*x*_Au_36−*x*_ nanoclusters, respectively.

## Graphical abstract

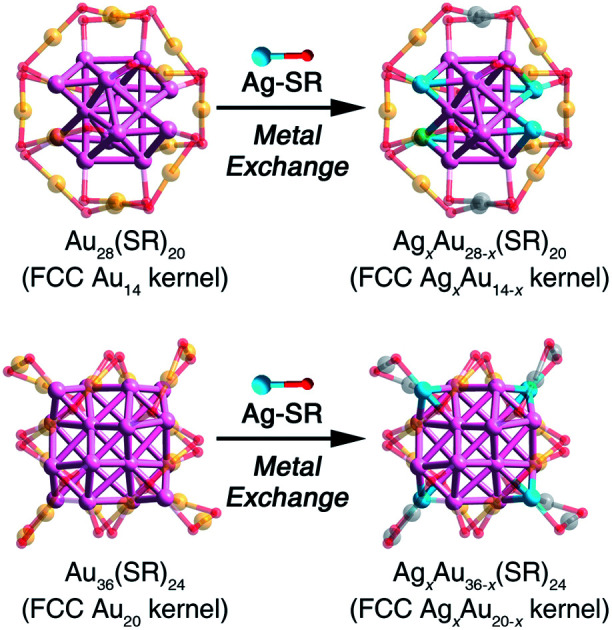

Doped nanoclusters provide an ideal model for understanding the metal synergy effect at the atomic level.^1–14^ However, doping a specific number of hetero-metal atoms at specific positions is a great challenge in the synthesis of doped metal nanoclusters with maintained structure.^15–19^ Metal displacement reactions based on standard electrode potentials are widely used in metal extraction and alloying. In modern chemistry, this method can effectively construct alloy nanoparticles with maintained structure, *i.e.*, reacting silver nanoparticles with gold salt can produce silver–gold alloy nanoparticles.^20–22^ Interestingly, when the size of metal nanoparticles decreases to the range of 1–2 nm, the nanoparticles exhibit molecular-like behaviour due to the strong quantum size effect, instead of metal behaviour in large nanoparticles (>3 nm).^23–30^ More precisely, the metal nanoparticles at 1–2 nm are non-metallic.

Previous studies indicated that metal replacement reactions can also work in these nanoclusters. For example, silver–gold alloy nanoclusters can be synthesized by the reaction of homo-silver nanoclusters with Au(i) salt. Reacting the Ag_25_ or Ag_29_ with Au(i) salt can produce Au_*x*_Ag_25−*x*_ and Au_*x*_Ag_29−*x*_ alloy nanoclusters.^31,32^ Interestingly, we previously reported a metal-exchange strategy by doping the template gold nanocluster with the thiolated metal complexes as the heteroatom source.^2,19^ Since it is hard to obtain metal nanoclusters composed of active metals, this strategy has been widely used in the synthesis of alloy nanoclusters, which not only provides an effective strategy for doping specific atoms into specific positions with controllable numbers, but also enhances the properties of alloy nanoclusters relative to the parent nanoclusters, such as fluorescence,^33–35^ catalytic activity,^36^ and chiral optical activity.^37^ Starting from the structure maintained, the synergistic effect can be understood at the atomic level. Moreover, the reversible metal-exchange process (*e.g.*, Au_25_ + Ag(i) ↔ Ag_*x*_Au_25−*x*_ + Au(i)) indicates that this process is not subject to the electrochemical potential constraints.^2,19^ However, the mechanism of this metal-exchange process has not been elucidated.

Herein, two fcc-structured ultra-small alloy nanoclusters which contain anisotropic metal cores, *i.e.*, Ag_*x*_Au_36−*x*_(SR)_24_ and Ag_*x*_Au_28−*x*_(SR)_20_ have been synthesized by the metal-exchange strategy on the previously reported Au_36_(SR)_24_ and Au_28_(SR)_20_ nanoclusters. In addition to isotropic icosahedral metal kernels, the understating of alloying anisotropic fcc metal kernels hopefully provides us more detailed information on the metal exchange. The single crystal X-ray diffraction results reveal that only specific gold atoms of Au_36_(SR)_24_ and Au_28_(SR)_20_ (*i.e.*, Au atoms at vertex sites in the metal core or in the motifs which are bonded with the vertex sites) can be exchanged by Ag atoms. Theoretical analysis of positive electrostatic potential (ESP) and population indicates that the metal-exchange process at vertex sites of the metal core is an electrophilic reaction. Specifically, the gold atoms with the lowest ESP and dual descriptor values could be exchanged with the silver atoms, which is similar to the vertex effect in large gold nanoparticles.

The fcc-structured Au_36_ and Au_28_ nanoclusters were synthesized by ligand-exchange on Au_38_ and Au_25_ nanoclusters with TBBT, respectively.^38,39^ The configuration of these nanoclusters can be viewed as the fcc-structured metal cores surrounding by SR–Au–SR complexes. In these metal cores, four vertex gold atoms in both Au_36_ and Au_28_ nanoclusters (Fig. 1) are observed. It is worth noting that our group previously reported the crystal structure of directly synthesized Ag_*x*_Au_36−*x*_ nanoclusters, which showed that the silver can only be doped on the motifs.^38^ We then carefully re-refined the model by substitutional disorder rather than positional disorder; the newly obtained results (Fig. S1 and S2†) indicate that the Ag occupancy sites are similar to the model in this work. We apologize for the wrong results due to the lack of experience in the treatment of alloy clusters at the early stage of our study in this area.^41^

**Fig. 1:**
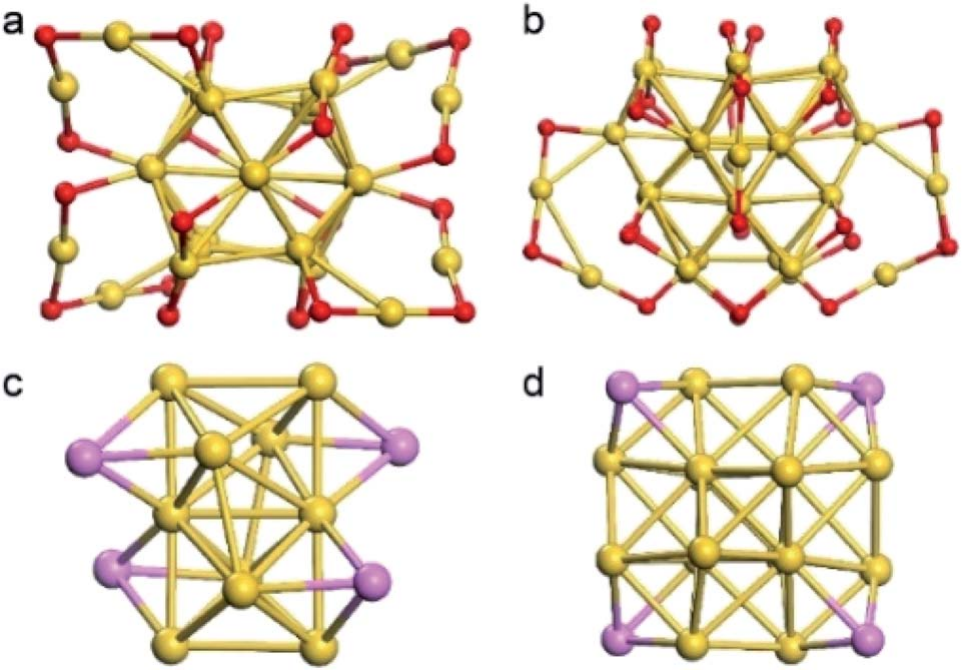
Total structure of Au_28_(SR)_20_ and Au_36_(SR)_24_ nanoclusters. (a) total structure of Au_28_(SR)_20_ nanoclusters; (b) total structure of Au_36_(SR)_24_ nanoclusters; (c) the Au_14_ metal core of Au_28_(SR)_20_ nanoclusters; (d) the Au_20_ metal core of Au_36_(SR)_24_ nanoclusters. Color label: yellow, Au; red, S; pink, Au at the vertex side of the metal core. Note that the C and H atoms are omitted for clarity.

In order to study the metal exchange behaviour in fcc-structured gold nanoclusters, Ag(i)–SR (where SR = TBBT) is used as the heterometal source. Besides, UV/Vis and ESI-TOF-MS spectroscopy methods are applied to monitor the metal exchange reaction. As shown in Fig. 2a and b, the peaks of homo-gold Au_28_ and Au_36_ nanoclusters exhibit continuous broadening with increasing amounts of Ag(i)–SR. It should be noted that the isosbestic point at 1.95 eV and multiple peaks at 1.9, 2.25 and 2.75 eV are found in Au_28_ doping, which implies that the metal exchange is a high yielding process. Meanwhile, the HOMO–LUMO gap decreases from 1.7 to 1.3 eV with the addition of 8 equivalents of Ag(i)–SR for Au_28_ and from ∼1.7 to ∼1.5 eV for Au_36_. The decreased HOMO–LUMO transition has also been reported in other silver doped gold nanoclusters. Furthermore, ESI-TOF-MS spectra illustrate a distribution of peaks after the addition of the Ag(i)–SR complex (Fig. 2c and d). The mass separation (89 Da) between peaks is equal to the difference between gold and silver atoms (that is, *M*_Au_ − *M*_Ag_ = 89 Da), demonstrating that silver has been successfully doped into the fcc-structured Au_28_ and Au_36_ nanoclusters. Specifically, after the addition of 8 equivalents of the Ag(i)–SR complex, up to ∼8 and ∼6 silver atoms could be found in the alloyed Au_28_ and Au_36_ nanoclusters, respectively.

**Fig. 2:**
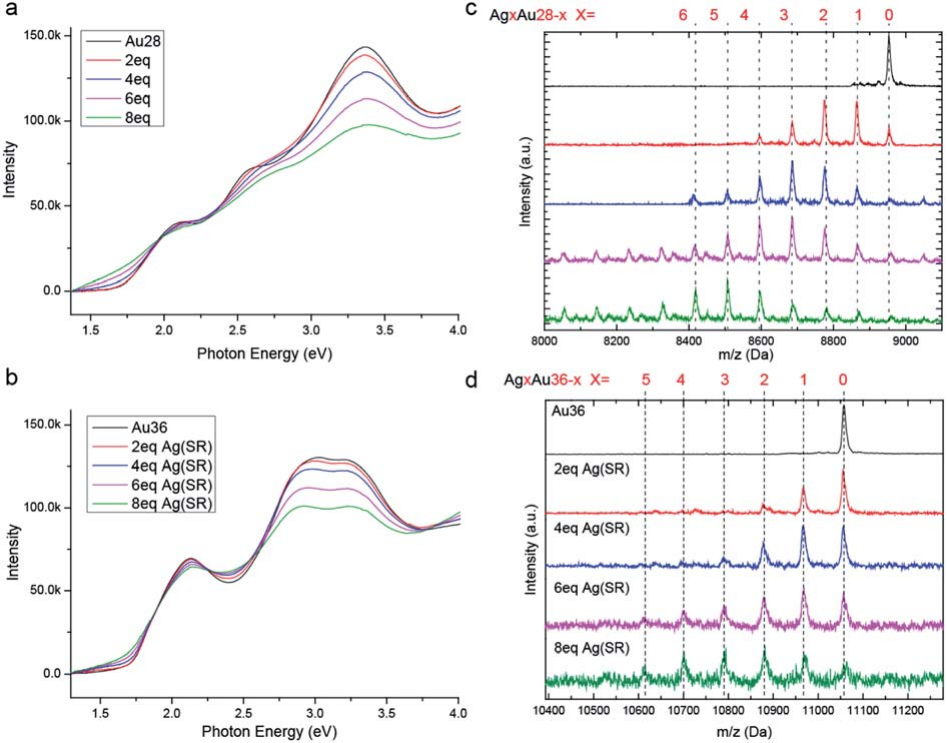
Time dependent photoelectron spectroscopy and electron spray ionization mass (ESI-MS) spectra of doping progress. (a and b) Time dependent photoelectron spectroscopy of Au_28_(TBBT)_20_ and Au_36_(TBBT)_24_ nanoclusters with different amounts of Ag(TBBT) complex, respectively; (c and d) time dependent ESI-MS spectra of Au_28_(TBBT)_20_ and Au_36_(TBBT)_24_ nanoclusters with different amounts of Ag(TBBT) complex, respectively.

X-ray crystallography was performed to ascertain the precise doping sites of silver atoms. Both nanoclusters were crystalized in mixed toluene/methanol, followed by X-ray diffraction. Data refinements involving partial occupancy were used to locate the Ag atoms (see detailed information in the ESI†). Note that for the doped Ag_*x*_Au_28−*x*_ nanocluster (*i.e.*, *x* > 3), it is hard to obtain single crystals with high quality. Thus, the less doped product, Ag_*x*_Au_28−*x*_ with *x* ∼ 1, was exploited. On the other hand, single crystals of Ag_*x*_Au_36−*x*_ (*x* ∼ 4) with high quality were obtained. Very recently, the structure of Ag_*x*_Au_36−*x*_ has been re-solved, and the doping sites of Ag heteroatoms are similar to the results in the current work.^40^

The detailed occupancy results of the doped Au_28_ nanocluster are shown in Fig. 3a and c, and the doping sites are highlighted in magenta in the core and green in the motif, respectively. The occupancy results clearly indicate that silver can exchange gold atoms at the vertex sites of the metal core (labelled 5, 5′, 8, and 8′), while Au in other sites cannot be exchanged. The average Ag atom found in the metal core is about 0.60. Meanwhile, one site in the motif which is bonded with the vertex site can be occupied by silver (not the site which is next to the vertex site). The average Ag occupancy in the motif is about 8.7%. Accordingly, the total Ag number in the doped Au_28_ nanocluster is about 0.95, which is consistent with the ESI-MS results (Fig. S3†).

**Fig. 3:**
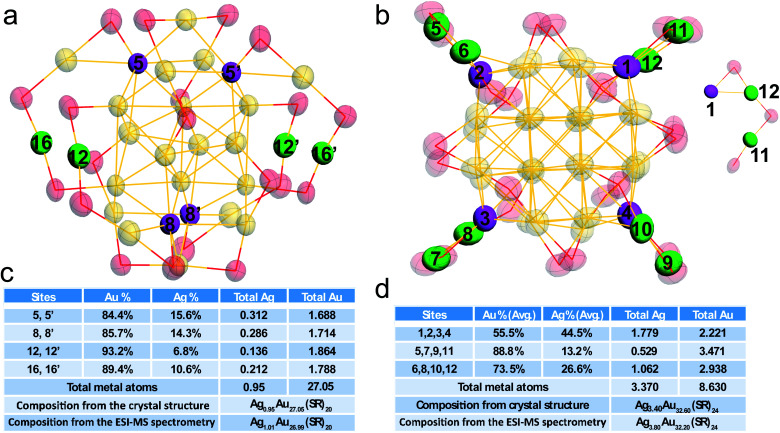
Total structure and occupancy information on silver doped Ag_*x*_Au_28−*x*_ and Ag_*x*_Au_36−*x*_ nanoclusters. (a) Ag_*x*_Au_28−*x*_; (b) Ag_*x*_Au_36−*x*_; detailed occupancy information of silver in doped Ag_*x*_Au_28−*x*_ (c) and Ag_*x*_Au_36−*x*_ (d) nanoclusters. The molecular structure of both nanoclusters with thermal ellipsoids set at the 50% probability level. All C and H atoms are omitted for clarity.

Fig. 3b and d show the detailed occupancy of the alloyed Au_36_ nanocluster. Similar to the doped Au_28_ nanocluster, silver can only substitute the gold atoms at the vertex sites of the Au_20_ core. The average Ag atom existent in the metal core is about 2.30. Meanwhile, the Au positions in the Au_2_(SR)_3_ motif (linked with the vertex sites) can also be occupied by silver atoms. It is worth noting that the probability of silver atoms at sites 6, 8, 10, and 12 which are close to the vertex site in the metal core (∼27.6%) is much smaller than that at sites 5, 7, 9, 11 (∼59.9%). Because of the similar chemical environments of these two sites, the probability difference is quite interesting.

Compared with the average doping of silver atoms in the isotropic Au_25_(SR)_18_ nanocluster, the Ag doping into the anisotropic, fcc-structured Au_28_ and Au_36_ nanoclusters is quite different. More importantly, a comparison of isotropic and anisotropic doping provides an opportunity for deep understanding of the metal exchange process at the atomic level. In this context, DFT calculations were carried out to find why the Ag atoms prefer to replace the Au atoms at the vertex sites of the fcc kernel. Brinck *et al.* found that the intriguing effects of nanostructures in gold catalysis can be explained by the appearance of positive electrostatic potential (ESP),^42^ and the ESP played an important role in the prediction of reaction sites. In this work, we analysed the distribution of the electrostatic potential, which is a well-established tool for analysing the chemical bonding and inter-molecular interaction. In order to reveal the substitution mechanism of exchanging the Au atoms on the vertexes in the Au_28_ and Au_36_ cores with Ag, the ESP of Ag(TBBT) was first calculated (Fig. 4A). The results indicate that the maximum value of ESP appears at the Ag atom site (47.60 kcal mol^−1^), and the smallest value appears at the S atom site (−30.28 kcal mol^−1^). This is due to the strong electronegativity of the S atom that leads to the polarization of the S–Ag bond, which makes electrons distribute around the S atom. Therefore, the Ag atom possesses the characteristics of electrophilic reaction sites.

**Fig. 4:**
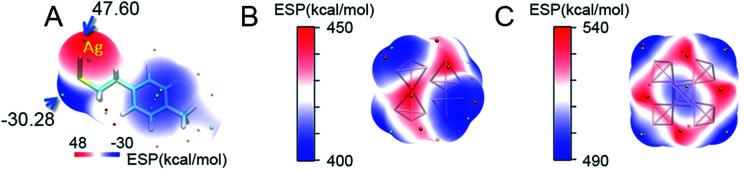
The surface electrostatic potential (ESP) of (A) Ag(TBBT) complex; (B) kernel of Au_28_; (C) kernel of Au_36_. The *tert*-butyl is replaced by methyl for convenience of calculation. Color scale bar: from blue to red, the value of ESP is increasing. Color balls: orange, the maximal value point of ESP; cyan, the minimum value point of ESP.

The calculations on the surface ESP of fcc cores in the two nanoclusters were also performed. For the Au_28_ nanocluster, it is interesting that the four vertex sites exhibit minimum ESP values (Fig. 4B). Similar to the Au_28_ nanocluster, the minimum ESP values for the Au_36_ nanocluster are also found near to the four vertexes of the Au kernel. Besides, another two minimum points are found within the gold kernel of Au_36_, which are arranged in the centre of the metallic kernel (Fig. 4C).

Based on these analyses, one may find that all of the vertex sites of the two gold cores possess minimum ESP values, demonstrating the different properties of the vertex site in comparison to other kernel positions. The population analysis of each kernel atom in three valence states (−1, 0, and +1) is further carried out on these nanoclusters. Based on the Hirshfeld charge results, the possibility of each atom's electrophilic or nucleophilic reaction is analysed by using the condensed Fukui function and condensed dual descriptor.^43,44^

As shown in Fig. 5A and C, to the Au_14_ kernel of Au_28_ nanoclusters, the Δ*f* values of the four vertex Au sites are all negative, indicating that these four sites are more prone to the electrophilic reaction. This result is consistent with the analysis of the surface ESP. Furthermore, Au_36_ (Fig. 5B and D) has four Au atoms (*i.e.*, Au_4_, Au_7_, Au_9_ and Au_13_) with minimum Δ*f* (about −0.0164) that do not occupy the vertex site; however, because the ESP values near the four Au atoms are higher than those of the vertexes, the electrophilic reaction is difficult to occur at these four sites. In contrast, the four Au atoms (*i.e.*, Au_11_, Au_18–20_) in the vertex sites not only satisfy the conditions of electrophilic reaction (Δ*f* < 0, about −0.0112), but also have smaller surface ESP. Accordingly, these four locations are more favorable for the occurrence of the electrophilic reaction.

**Fig. 5:**
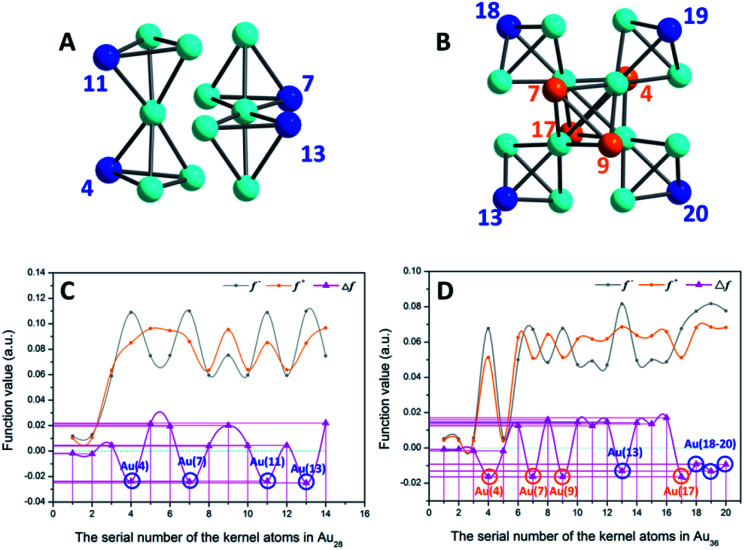
(A and B) Kernel structures of Au_28_ and Au_36_ clusters respectively. (C and D) Fukui function (*f*^−^ and *f*^+^)^43^ and dual descriptor (Δ*f*)^44^ values of whole kernel atoms. The comparison expression between the Fukui function and dual descriptor is Δ*f* = *f*^+^ − *f*^−^. Blue balls, atoms at vertex sites; orange and turquoise balls, other Au atoms on the kernel.

In addition, we also calculated the ESP of the kernels of the Au_23_, Au_24_, and Au_25_ clusters and the Δ*f* for each kernel atom. As shown in Fig. S3,† the ESP at the apex of the cores of Au_23_ and Au_24_ clusters is significantly smaller than other regions, which indicates that the atoms at the vertex are relatively more prone to electrophilic substitution reactions. In other words, based on the metal exchange mechanism proposed in this paper, these atoms at the vertex are easily replaced by Ag. In fact, the previous work reported that Ag doped Au_23−*x*_Ag_*x*_(SR)_16_^−^ was doped at exactly the apex.^45^ Fig. S3C† shows the ESP of the core of the Au_25_ cluster. The ESP of the atoms on the surface of the icosahedron is significantly smaller than that of the central atom. In order to further clarify the reaction mechanism of the atoms at the vertices of the cluster kernel described above, the calculation of Δ*f* was also implemented. As shown in Table S3,† the Δ*f* at the core vertex of Au_23_ and Au_24_ is negative, indicating that these sites are more prone to electrophilic substitution reactions. However, the Δ*f* of the core atom of the Au_25_ symmetry Au is not so symmetrical, which needs further exploration.

Through combined analyses of the surface electrostatic potential and the dual descriptor, it can be concluded that the sites of the vertexes in the fcc kernels can be subject to electrophilic reactions.

## Conclusions

In this work, we have succeeded in the synthesis of Ag_*x*_Au_28−*x*_(SR)_20_ and Ag_*x*_Au_36−*x*_(SR)_24_ nanoclusters by metal exchange of the Au_28_ and Au_36_ nanoclusters with the Ag(i)SR complex. The mechanism of the metal exchange process, which does not conform to the redox sequence, has been mapped out by single crystal X-ray diffraction and DFT calculations. Interestingly, in the nanocluster range, the metal replacement reaction does not follow the redox sequence; instead, the quantum confinement effect induces electronic density concentration at vertex sites of the metal core, which leads to the electrophilic reaction.

## Conflicts of interest

There are no conflicts to declare.

## Supplementary Material

NA-002-C9NA00746F-s001

NA-002-C9NA00746F-s002
