# Supplementary material for: The mechanism of metal exchange in non-metallic nanoclusters
Source: Nanoscale Adv. 2020 Jan 14;2(2):664–8. doi: 10.1039/c9na00746f (PMC9419833; doi:10.1039/c9na00746f)
Supplement: NA-002-C9NA00746F-s001 [file NA-002-C9NA00746F-s001.pdf]

Electronic Supplementary Material (ESI) for Nanoscale Advances.
This journal is © The Royal Society of Chemistry 2020

Mechanism of Metal Exchange in Non-metallic Nanoclusters

Shuxin Wang,[a,*] Lin Xiong,[b] Guodong Sun,[a] Li Tang,[a] Jun Zhang,[c] Yong Pei,[b,*] Manzhou Zhu[a,*]

a. Department of Chemistry and Center for Atomic Engineering of Advanced Materials, Anhui University, Hefei, Anhui 230601, PR China.
b. Department of Chemistry, Key Laboratory of Environmentally Friendly Chemistry and Applications of Ministry of Education, Xiangtan University, Xiangtan, Hunan 411105, PR China.
c. School of Materials and Chemical Engineering, Anhui Jianzhu University, Hefei, Anhui 230601, PR China.

1. Experimental

Materials.

Unless specified, reagents were purchased from ACROS Organics or Sigma-Aldrich and used without further purification. Phenylethyl mercaptan (PET, ≥99%), 4-*tert*-Butylbenzenethiol (TBBT, 97%) Tetrachloroauric(III) acid ($HAuCl_4 \cdot 3H_2O$, >99.99% metals basis), $AgNO_3$ (99.85%), $NaBH_4$ (>98%) were received from ACROS Organic. Toluene (HPLC grade, ≥99.9%, Sigma-Aldrich), Ethanol (HPLC grade, ≥99.9%), Methanol (HPLC grade, ≥99.9%), and Methylene chloride (HPLC grade, ≥99.9%) were from Sigma-Aldrich. Pure water was ordered from Wahaha Co LTD.

Synthesis of Ag(TBBT)

1 mL aqueous solution of $AgNO_3$ (0.21 mM) was added into 7 mL ethanol solution which contain 0.6 mL TBBT and 2 mL tri-ethylamine under vigorous stirring. After 30 mins stir-mixing, the contents were taken to a centrifuge tube and centrifuged at ~6500 rpm. The solution was then removed and the precipitate was washed several times with ethanol/water to remove the redundant thiol ligands. The Ag(TBBT) (white solid) was obtained.

Synthesis of $Au_{28}(TBBT)_{20}$

The synthesis steps were similar to a previously reported process but with some minor modifications.[S1] First step: Synthesis of $Au_{25}(SR)_{18}^{-}TOA^{+}$ nanocluster, which according to Murray's previously reported work.[S2] Second step: Synthesis of $Au_{28}(TBBT)_{20}$ nanocluster. i) 20 mg $Au_{25}(SR)_{18}^{-}TOA^{+}$ nanocluster was dissolved in ~2 mL toluene, then 1 mL of TBBT was added into the solution. After that, the reaction temperature was quickly arising to ~80 ℃. The reaction was continued for the next 2 hours under vigorous stirring. The crude product was washed with methanol for 3 times to remove the excessed thiol ligands and by-products. The obtained nanoclusters were crystalized in mix solvent of toluene and methanol. Black crystals were observed after about one week.

Synthesis of $Au_{36}(TBBT)_{24}$

The synthesis steps were similar to a previously reported process but with some minor modifications.[S3] Frist step: $Au_{38}(PET)_{24}$ nanoclusters were prepared by the reported size focusing method. Second step: ~20 mg $Au_{38}(PET)_{24}$ nanoclusters were dissolved in ~1 mL toluene which containing ~1 mL TBBT. The reaction temperature was quickly arising to 80 ℃, and the reaction was continued for the next 12 hours under vigorous stirring. The crude produce was washed with methanol for at least three times to remove the excessed thiol ligands and byproducts. The obtained nanoclusters were crystalized in mix

solvent of toluene and methanol. Dark green crystals were observed after about one week.

Reaction of Ag(TBBT) with $Au_{28}(TBBT)_{20}$ nanocluster

30 mg $Au_{28}(TBBT)_{20}$ nanocluster (3.4 x 10^{-3} mmol) was dissolved in 10 mL toluene, and ~8 equivalent of Ag(TBBT) (~7.4 mg powder, 2.7 x10^{-2} mmol) salt was added for four times, stir for 15 minutes for each addition.

Reaction of Ag(TBBT) with Au36(TBBT)24 nanocluster

30 mg $Au_{36}(TBBT)_{24}$ nanocluster (2.7 x 10^{-3} mmol) was dissolved in 10 mL toluene, and ~8 equivalent of Ag(TBBT) (~5.8 mg powder, 2.2 x 10^{-2} mmol) salt was added for four times, stir for 15 minutes for each addition.

Spectroscopic studies

UV-Vis absorption spectra were obtained from an Agilent 8453 instrument, and DCM was used as solvent for experiments. ESI MS was recorded by a Waters Q-TOF mass spectrometer equipped with Z-spray source. The source temperature was kept at 70 °C. The sample was directly infused into the chamber at 5 μL/min. The spray voltage was kept at 2.20 kV and the cone voltage at 60 V. To prepare the ESI sample, clusters were dissolved in toluene (1 mg/mL) and diluted (v/v=1:2) by dry methanol containing 5 mM CsOAc to ionize the clusters by forming Cs^+-cluster adducts.

X-ray crystallographic analysis.

The data collections for single crystal X-ray diffraction was carried out on a Bruker Smart APEX II CCD diffractometer at 170 K equipped with a MoKα radiation (λ = 0.71073 Å). Data reductions and absorption corrections were performed using the SAINT and SADABS programs, respectively.[S4] The structure was solved by direct methods and refined with full matrix least squares on F^2 using OLEX2 with SHELXT/XL software package.[S5,S6] All non-hydrogen atoms were refined anisotropically, and all the hydrogen atoms were set in geometrically calculated positions and refined isotropically using a riding model.

Computational methods

All calculations were performed using the Gaussian09 software package[S7] at the theoretical level of PBE0/Def2TZVP. Note that the implementation of geometric optimization is not limited by symmetry, and all convergence parameters are default. The quantitative analysis of Hirshfeld atomic charge, molecular surface electrostatic potential (ESP), Fukui function and dual descriptors are all carried out by Multiwfn, version 3.5.[S8] Among them, the color map of the molecular surface ESP is drawn by the VMD software.

Ref.

S1. C. Zeng, T. Li, A. Das, N. L. Rosi, R. Jin, *J. Am. Chem. Soc.*, 2013, **135**, 10011.

S2. J. F. Parker, J. E. F. Weaver, F. McCallum, C. A. Fields-Zinna, R. W. Murray, *Langmuir*, 2010, **26**, 13650.

S3. C. Zeng, H. Qian, T. Li, G. Li, N. L. Rosi, B. Yoon, R. N. Barnett, R. L. Whetten, U. Landman, R. Jin, *Angew. Chem., Int. Ed.*, 2012, **51**, 13114.

S4. SMART, and SADABS, Bruker AXS Inc., Madison, Wisconsin, USA.

S5. G. M. Sheldrick, *Acta Crystallogr. Sect. A* 2008, **64**, 112.

S6. O. V. Dolomanov, L. J. Bourhis, R. J. Gildea, J. A. K. Howard, H. Puschmann, *J. Appl. Cryst.*,

2009, **42**, 339.
S7. Gaussian 09, Revision D.01, M. J. Frisch, G. W. Trucks, H. B. Schlegel, G. E. Scuseria, M. A. Robb, J. R. Cheeseman, G. Scalmani, V. Barone, B. Mennucci, G. A. Petersson, H. Nakatsuji, M. Caricato, X. Li, H. P. Hratchian, A. F. Izmaylov, J. Bloino, G. Zheng, J. L. Sonnenberg, M. Hada, M. Ehara, K. Toyota, R. Fukuda, J. Hasegawa, M. Ishida, T. Nakajima, Y. Honda, O. Kitao, H. Nakai, T. Vreven, J. A. Montgomery, Jr., J. E. Peralta, F. Ogliaro, M. Bearpark, J. J. Heyd, E. Brothers, K. N. Kudin, V. N. Staroverov, T. Keith, R. Kobayashi, J. Normand, K. Raghavachari, A. Rendell, J. C. Burant, S. S. Iyengar, J. Tomasi, M. Cossi, N. Rega, J. M. Millam, M. Klene, J. E. Knox, J. B. Cross, V. Bakken, C. Adamo, J. Jaramillo, R. Gomperts, R. E. Stratmann, O. Yazyev, A. J. Austin, R. Cammi, C. Pomelli, J. W. Ochterski, R. L. Martin, K. Morokuma, V. G. Zakrzewski, G. A. Voth, P. Salvador, J. J. Dannenberg, S. Dapprich, A. D. Daniels, O. Farkas, J. B. Foresman, J. V. Ortiz, J. Cioslowski, and D. J. Fox, Gaussian, Inc., Wallingford CT, 2013.
S8. T. Lu, F. Chen, *J. Comput. Chem.*, 2012, **33**, 580.

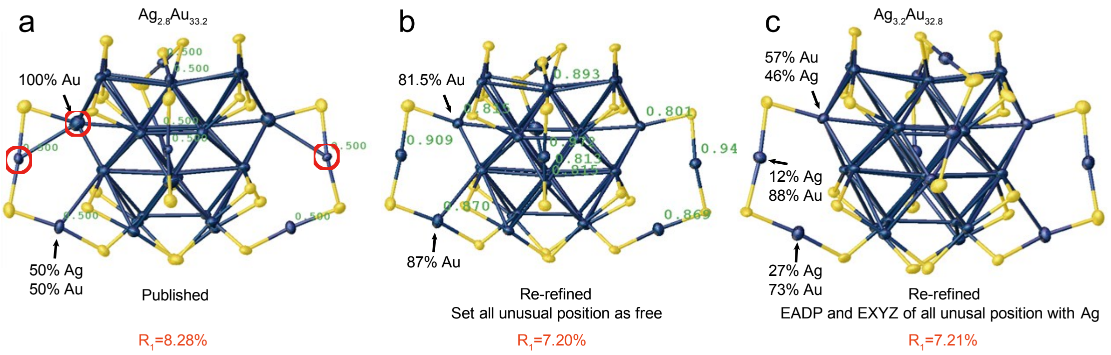

Figure S1. Re-refined model based on published results. a) The abnormal thermodynamic ellipsoid found in the published work; b) re-refined model based on the published results. Note, in this model, we set all of the metal atoms as gold, then set the occupy as free for abnormal positions. The R_1 value was decreased from 8.28% to 7.20%. This result indicate that the electronic density in this position is less than gold; c) Substitutional disorder operation based on b). The results indicate silver atoms are not only replace the gold atoms at the motif positions, but also replace the gold atoms at the metal kernel.

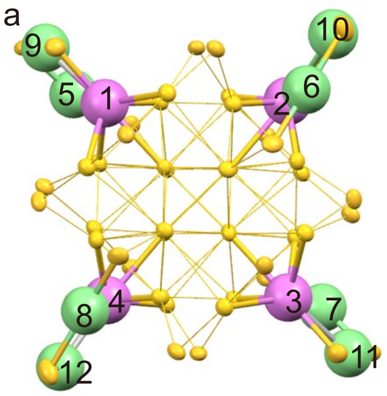

b

Sites	Au%	Ag%
1	53.8%	46.2%
2	54.1%	45.9%
3	54.3%	45.7%
4	55.8%	44.2%
5	73.4%	26.6%
6	76.5%	23.5%
7	75.9%	24.1%
8	82.8%	17.2%
9	87.9%	12.1%
10	84.1%	15.9%
11	92.8%	7.2%
12	85.8%	14.2%

Figure S2. a) The re-modelled structure of $Ag_xAu_{36-x}(TBBT)_{24}$ nanocluster based on the published results;[ref. 38] b) the occupy information of silver doped $Ag_xAu_{36-x}(TBBT)_{24}$ nanocluster based on the published results.[ref. 38]

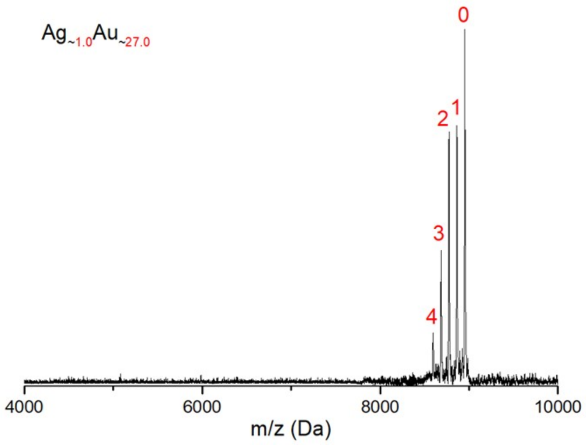

Figure S3. ESI-MS spectrum of $Ag_xAu_{28-x}(TBBT)_{20}$ crystals dissolved in toluene (1 mg/mL) and diluted (v/v=1:2) by dry methanol containing 5 mM CsOAc. The results reveal the average of silver atom number is ~1.

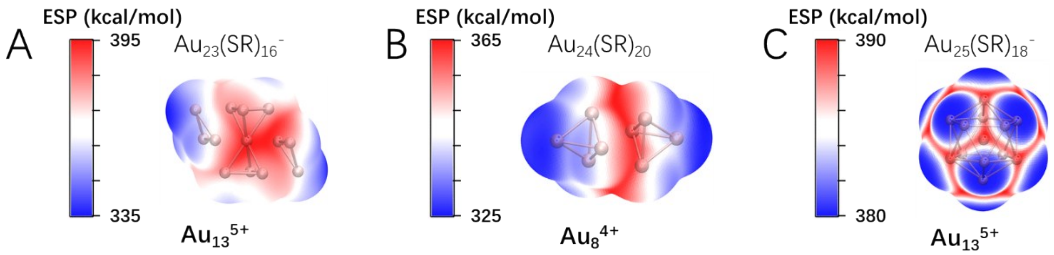

Figure S4. The surface electrostatic potential (ESP) of A) kernel of Au_{23}; B) kernel of Au_{24}; C) kernel of Au_{25}. Color scale bar: From blue to red, the value of ESP is increasing.

Table S1 Crystal data and structure refinement for 1967149_28.

Identification code	1967149_28
Empirical formula	$C_{200}H_{259}Ag_{0.95}Au_{27.06}S_{20}$
Formula weight	8735.68
Temperature/K	170
Crystal system	monoclinic
Space group	C2/c
a/Å	40.4373(18)
b/Å	24.4078(11)
c/Å	32.506(3)
α/°	90
β/°	119.448(2)
γ/°	90
Volume/Å^3	27938(3)
Z	4
ρ_{calc}g/cm^3	2.077
μ/mm^{-1}	14.388
F(000)	15846.0
Radiation	MoKα (λ = 0.71073)
2Θ range for data collection/°	2.798 to 55.266
Index ranges	-51 ≤ h ≤ 52, -31 ≤ k ≤ 31, -42 ≤ l ≤ 32
Reflections collected	204214
Independent reflections	31707 [R_{int} = 0.0799, R_{sigma} = 0.0695]
Data/restraints/parameters	31707/2139/1216
Goodness-of-fit on F^2	1.074
Final R indexes [I>=2σ (I)]	R_1 = 0.0635, wR_2 = 0.1541
Final R indexes [all data]	R_1 = 0.1241, wR_2 = 0.1979
Largest diff. peak/hole / e Å^{-3}	4.75/-2.82

Table S2 Crystal data and structure refinement for 1967209_36.

Identification code	1967209_36
Empirical formula	$C_{240}H_{312}Ag_{3.36}Au_{32.64}S_{24}$
Formula weight	10757.86
Temperature/K	170
Crystal system	monoclinic
Space group	C2/c
a/Å	74.3145(17)
b/Å	20.8019(5)
c/Å	39.5827(8)
α/°	90
β/°	101.783(2)
γ/°	90
Volume/Å^3	59901(2)
Z	8
ρ_{calc}g/cm^3	2.386
μ/mm^{-1}	16.335
F(000)	38980.0
Crystal size/mm^3	0.24 × 0.22 × 0.2
Radiation	MoKα (λ = 0.71073)
2Θ range for data collection/°	2.036 to 56
Index ranges	-98 ≤ h ≤ 98, -27 ≤ k ≤ 27, -52 ≤ l ≤ 51
Reflections collected	467712
Independent reflections	72201 [R_{int} = 0.0596, R_{sigma} = 0.0420]
Data/restraints/parameters	72201/13/2763
Goodness-of-fit on F^2	1.012
Final R indexes [I>=2σ (I)]	R_1 = 0.0415, wR_2 = 0.0899
Final R indexes [all data]	R_1 = 0.0808, wR_2 = 0.0983
Largest diff. peak/hole / e Å^{-3}	1.88/-1.19

Table S3. Fukui function ($f^{(-)}$ and $f^{(+)}$) and dual descriptor ($\triangle f$) values of kernel atoms of Au_{23}, Au_{24} and Au_{25}. The inset is a schematic representation of the core of three clusters. The $\triangle f$ of the atoms in the circles is large negative value, indicating that the electrophilic substitution reaction is easy to occur at these positions.

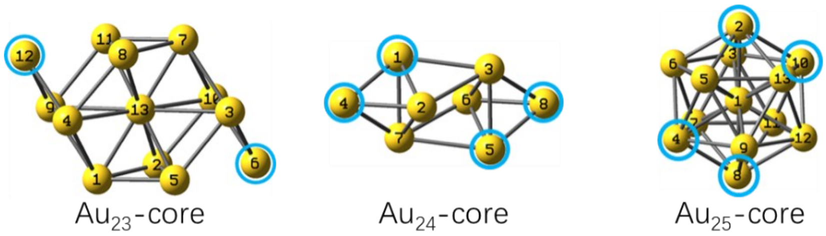

		Hirshfeld charges			$f^{(-)}$	$f^{(+)}$	$\triangle f$
		N	N-1	N+1			
Au_{23}-core	Au1	0.353	0.426	0.291	0.072	0.062	**-0.011**
	Au2	0.416	0.474	0.315	0.059	0.101	**0.042**
	Au3	0.389	0.477	0.311	0.088	0.078	**-0.01**
	Au4	0.373	0.455	0.293	0.082	0.08	**-0.002**
	Au5	0.399	0.456	0.309	0.056	0.091	**0.034**
	Au6	0.519	0.651	0.436	0.132	0.083	**-0.049**
	Au7	0.353	0.426	0.291	0.072	0.062	**-0.011**
	Au8	0.416	0.474	0.315	0.059	0.101	**0.042**
	Au9	0.389	0.477	0.311	0.088	0.078	**-0.01**
	Au10	0.373	0.455	0.293	0.082	0.08	**-0.002**
	Au11	0.399	0.456	0.309	0.056	0.091	**0.034**
	Au12	0.519	0.651	0.436	0.132	0.083	**-0.049**
□	Au13	0.1	0.123	0.088	0.022	0.012	**-0.01**
Au_{24}-core	Au1	0.478	0.604	0.393	0.126	0.085	**-0.041**
	Au2	0.461	0.573	0.311	0.112	0.15	**0.038**
	Au3	0.447	0.557	0.289	0.109	0.158	**0.049**
	Au4	0.614	0.766	0.507	0.153	0.107	**-0.046**
	Au5	0.478	0.604	0.393	0.126	0.085	**-0.041**
	Au6	0.461	0.573	0.311	0.112	0.15	**0.038**
	Au7	0.447	0.557	0.289	0.109	0.158	**0.049**
□	Au8	0.614	0.766	0.507	0.153	0.107	**-0.046**
Au_{25}-core	Au1	0.134	0.128	0.13	-0.006	0.003	**0.01**
	Au2	0.406	0.491	0.338	0.086	0.067	**-0.018**
	Au3	0.405	0.489	0.309	0.084	0.096	**0.013**
	Au4	0.406	0.491	0.354	0.086	0.052	**-0.034**
	Au5	0.406	0.488	0.312	0.082	0.093	**0.011**
	Au6	0.406	0.488	0.312	0.082	0.093	**0.011**
	Au7	0.405	0.489	0.309	0.084	0.096	**0.013**
	Au8	0.406	0.491	0.338	0.086	0.067	**-0.018**
	Au9	0.405	0.489	0.309	0.084	0.096	**0.013**
	Au10	0.406	0.491	0.354	0.086	0.052	**-0.034**

	Au11	0.406	0.488	0.312	0.082	0.093	**0.011**
	Au12	0.406	0.488	0.312	0.082	0.093	**0.011**
□	Au13	0.405	0.489	0.309	0.084	0.096	**0.013**
